# Neutral Effects of Combined Treatment With GLP-1R Agonist Exenatide and MR Antagonist Potassium Canrenoate on Cardiac Function in Porcine and Murine Chronic Heart Failure Models

**DOI:** 10.3389/fphar.2021.702326

**Published:** 2021-07-26

**Authors:** Evelyne J. Demkes, Steven Wenker, Max J. M. Silvis, Martijn M. J. van Nieuwburg, M. Joyce Visser, Marlijn S. Jansen, Maike A. D. Brans, Evelyn Velema, Joost P. G. Sluijter, Imo E. Hoefer, Dominique P. V. de Kleijn, Leo Timmers, Saskia C. A. de Jager

**Affiliations:** ^1^ Department of Cardiology, Laboratory of Experimental Cardiology, University Medical Center Utrecht, Utrecht, Netherlands; ^2^ UMC Utrecht Regenerative Medicine Center, Circulatory Health Laboratory, University Utrecht, University Medical Center Utrecht, Utrecht, Netherlands; ^3^ Department of Cardiology, University Medical Center Utrecht, Utrecht, Netherlands; ^4^ Central Diagnostic Laboratory, University Medical Center Utrecht, Utrecht, Netherlands; ^5^ Department of Vascular Surgery, University Medical Centre Utrecht, Utrecht, Netherlands; ^6^ Department of Cardiology, St. Antonius Hospital, Utrecht, Netherlands

**Keywords:** chronic heart failure, glucagon-like peptide-1 agonist, mineralocorticoid receptor antagonist, porcine, mouse, myocardial infarction, cardiac function

## Abstract

**Background:** Ischemia-reperfusion and cardiac remodeling is associated with cardiomyocyte death, excessive fibrosis formation, and functional decline, eventually resulting in heart failure (HF). Glucagon-like peptide (GLP)-1 agonists are reported to reduce apoptosis and myocardial infarct size after ischemia-reperfusion. Moreover, mineralocorticoid receptor antagonists (MRAs) have been described to reduce reactive fibrosis and improve cardiac function. Here, we investigated whether combined treatment with GLP-1R agonist exenatide and MRA potassium canrenoate could minimize cardiac injury and limit HF progression in animal models of chronic HF.

**Methods and Results:** Forty female Topigs Norsvin pigs were subjected to 150 min balloon occlusion of the left anterior descending artery (LAD). Prior to reperfusion, pigs were randomly assigned to placebo or combination therapy (either low dose or high dose). Treatment was applied for two consecutive days or for 8 weeks with a continued high dose *via* a tunneled intravenous catheter. Using 2,3,5-Triphenyltetrazolium chloride (TTC) staining we observed that combination therapy did not affect the scar size after 8 weeks. In line, left ventricular volume and function assessed by three-dimensional (3D) echocardiography (baseline, 7 days and 8 weeks), and cardiac magnetic resonance imaging (CMR, 8 weeks) did not differ between experimental groups. In addition, 36 C57Bl/6JRj mice underwent permanent LAD-occlusion and were treated with either placebo or combination therapy prior to reperfusion, for two consecutive days *via* intravenous injection, followed by continued treatment *via* placement of osmotic mini-pumps for 28 days. Global cardiac function, assessed by 3D echocardiography performed at baseline, 7, 14, and 28 days, did not differ between treatment groups. Also, no differences were observed in cardiac hypertrophy, assessed by heart weight/bodyweight and heart weight/tibia length ratio.

**Conclusion:** In the current study, combined treatment with GLP-1R agonist exenatide and MR antagonist potassium canrenoate did not show beneficial effects on cardiac remodeling nor resulted in functional improvement in a small and large animal chronic HF model.

## Introduction

Chronic heart failure (HF) incidence, most commonly originating from myocardial infarction (MI) is steadily increasing worldwide and remains a major cause of death (Schmidt et al., 2012). Oxygen and nutrient deprivation during MI induces apoptosis of cardiomyocytes. Reperfusion of the occluded coronary artery is essential to limit myocardial damage but is also responsible for ischemia-reperfusion (IR) injury (Yellon and Hausenloy, 2007). As a consequence, a progressive remodeling response is initiated, consisting of reactive fibrosis formation, hypertrophy, and contractile dysfunction, which eventually leads to chronic HF (Bhatt et al., 2017). To prevent progression towards chronic HF, development of novel therapeutics that prevent progressive remodeling is of major importance.

Exenatide is a human glucagonlike peptide-1 receptor (GLP-1R) agonist. GLP-1R agonists are known for the treatment of type 2 diabetes, but have also been shown to be cardio-protective in preclinical and clinical studies by reducing myocardial infarct size and improving ventricular function after ischemia (Noyan-Ashraf et al., 2009; Lonborg et al., 2012; Read et al., 2012). Moreover, in a large animal model of myocardial IR injury, Exenatide specifically reduced myocardial apoptosis, resulting in a smaller infarct size and improved cardiac function (Timmers et al., 2009). Mineralocorticoid receptor (MR) signaling has a critical role in the fibrotic response observed in adverse cardiac remodeling (Brilla and Weber, 1992) treatment with MR antagonists (MRAs) resulted in a significant decrease in interstitial fibrosis and improved left ventricular function in rodents after permanent MI (Wang et al., 2004). In addition, MRAs have proven beneficial effects on mortality and morbidity in patients with established HF (Pitt et al., 1999; Pitt et al., 2003; Zannad et al., 2011), suggesting MR signaling is involved in HF progression.

Combining promising therapies that target the underlying process of IR injury and cardiac remodeling could further minimize cardiac injury and limit progression to HF. Therefore, we aimed to evaluate the effect of combined therapy of GLP-1R agonist exenatide and MRA potassium canrenoate on cardiac function in the development of chronic HF. To establish this, we use a clinically relevant large animal model of severe IR injury and a mice model of permanent myocardial infarction.

## Methods

### Animals

All animal experiments were approved by the local animal welfare committee of the University Medical Center Utrecht and were conducted in accordance with the “Guide for the Care and Use of Laboratory Animals.” A total of 40 female Dalland Landrace pigs (69.2 ± 4.2 kg) (Topigs Norsvin, Van Beek SPF varkensfokkerij B.V., Lelystad, Netherlands) were used in this study. All animals were conventionally housed in stables with concrete floor and straw bedding with a light/dark cycle of 12 h and were fed a standard diet with water ad libitum. Rubber bite sticks were provided as environmental enrichment. Sample size calculation was based on end-diastolic volume (EDV) measured with cardiac magnetic resonance imaging (CRM) as the primary endpoint. Based on historical data, we expected a 25% improvement on EDV (165 ml with a sigma of 25 ml). This, together with a power of 90%, alpha of 0.05, and an expected post-operative mortality of 25%, resulted in a group size of 10 animals per group. In addition, a total of 36 male C57Bl/6JRj mice were used. All mice were conventionally housed in type III cages with filter top, Aspen Woodchip bedding, and a plastic shelter with light/dark cycles of 12 h and food and water ad libitum. Tissues were provided as environmental enrichment. Sample size calculation was based on end-systolic volume (ESV) as the primary endpoint. With a power of 90%, alpha of 0.05, estimated effect size of 40 μL difference in volume, standard deviation of 29 μL (based on historical data), and estimated post-operative mortality of 25% this resulted in a group size of 18 animals per group.

### Porcine Efficacy Study

#### Premedication, Anesthesia, and Analgesia

Pigs were pre-treated orally with amiodarone for 10 days (1200 mg loading dose, 800 mg/day maintenance), clopidogrel (75 mg/day) and acetylsalicylic acid (320 mg loading dose 7 days before the experiment, 80 mg/day maintenance). One day before surgery, animals received a buprenorphine patch (5 μg/h). On the day of surgery, anesthesia and analgesia was induced by intramuscular injection of ketamine (15 mg/kg), midazolam (0.75 mg/kg) and atropine (0.015 mg/kg) followed by intravenous (*i.v.*) administration of thiopental (4 mg/kg). Animals were intubated and mechanically ventilated with a 1:2 oxygen-air ratio. Continuous sedation was achieved with *i.v.* pancuronium (0.1 mg/kg/h), midazolam (0.4 mg/kg/h) and sufentanil (2.5 μg/kg/h).

#### Surgical Procedure and Intravenous Line Installation

Pigs were subjected to closed-chest LAD coronary artery balloon occlusion for 150 min. After arterial and venous access was obtained, a catheter (8FR JL4 guiding) was placed in the left coronary tree and a coronary angiogram was acquired. Afterward, the LAD diameter was measured and an adequately sized balloon was placed immediately after the first diagonal branch of the LAD. Balloon inflation and LAD occlusion was verified with a coronary angiogram, at the start, after 60 and 120 min occlusion time, and right before deflation of the balloon. After deflation of the balloon LAD passage was verified with a coronary angiogram. During the occlusion procedure, animals were defibrillated in case of ventricular fibrillation (VF) while receiving 150 mg amiodarone bolus *i.v.*, with a maximum of three boluses.

All animals received an *i.v.*-line from the jugular vein canalled to the back. The iv-line was flushed daily with 0.9% NaCl-solution followed by heparin-solution (0.1% heparin in 0.9% NaCl-solution) and a mesh bandage around the thorax to prevent the *i.v.*-line from being damaged. The tunneled catheter was closed for three out of four groups after 7 days and the external part was removed.

#### Treatment

Twenty min before reperfusion, pigs were randomly assigned to one of four treatment groups (Supplementary Table 1). Pigs received either placebo (2 ml saline) or exenatide/potassium canrenoate treatment (low dose, 0.05 μg/kg exenatide and 1 mg/kg of potassium canrenoate; high dose, 0.15 μg/kg exenatide and 1 mg/kg of potassium canrenoate; continuous high dose: 0.15 μg/kg exenatide and 1 mg/kg of potassium canrenoate) as two separate administrations *via* the ear-vein catheter. *I.v.* treatment continued 2 times daily for two consecutive days and one group continued 2 times daily treatment for entire follow-up period (continuous high dose).

Note: Animals treated with the continuous high dose were initially treated with subcutaneous (s.c.) injections (0.05 μg/kg exenatide and 2 mg/kg potassium canrenoate). However, twice daily injections caused significant distress to the animals. Therefore, after the third animal in this group, we decided to leave the tunneled catheter in place to proceed with intravenous administrations for the entire study duration. In these animals, none of the parameters studied differ from the other animals in the group.

#### Echocardiography

Before induction of ischemia, and 1 and 8 weeks after reperfusion, all pigs underwent transthoracic and 3D transesophageal echocardiography (3D-TEE) as previously described (Ellenbroek et al., 2016). In short, 3D-TEE images were made using a X7-2T transducer on an iE33 ultrasound device (Philips, Eindhoven, Netherlands). The pig was placed in the right lateral position and the echo probe was inserted for 50–60 cm in the esophagus. After selecting the 3D full volume option, mechanical ventilation was switched of temporarily to obtain good images. Images were analyzed with QLab 10.7 software (Philips, Eindhoven, Netherlands).

#### Cardiac Magnetic Resonance Imaging

Eight weeks after MI, porcine animals underwent cardiac magnetic resonance imaging (CRM) using a 1,5 T magnet device (Achieva, Philips Medical Systems, Netherlands) under general anesthesia, as described above. Animals were placed in a supine position and imaging was performed using a respiratory corrected, cardiac gated steady-state free precession (SSFP) cine sequence. CMR images were analyzed using Medvisio Segment (Medvisio, Lund, Sweden). Cardiac volumes and myocardial mass were determined by summation of ROIs of short axis slices covering the entire left ventricle multiplied by the slice thickness.

#### Infarct/Scar Size Determination

Ventricular fibrillation was induced by placing a 9V battery on the heart and pigs were sacrificed by exsanguination under anesthesia. The heart was excised and cut in 5 short-axis slices from apex to base. To discriminate between infarct tissue and viable tissue, slices were incubated in 1% pre-warmed 2,3,5-Triphenyltetrazolium chloride (TTC) (Sigma-Aldrich Chemicals, Zwijndrecht, Netherlands) in 0.9% NaCl at 37° for 15 min. Each slice was photographed at the apical and basal side in presence of a ruler and analyzed with ImageJ software (NIH, Bethesda, MD, United States).

#### Circulating Markers and Histological Analysis

Plasma samples were obtained at different time points after reperfusion by whole-blood centrifugation at 1850 *g* and immediately stored at −80°C. Troponin I levels were measured from samples 24 h after reperfusion using a clinical chemistry analyzer (AU5811, Beckman Coulter). For histological analysis, infarcted myocardial tissue was processed, paraffin-imbedded and cut into 5 µm sections after conserved in 4% paraformaldehyde for at least 7 days. Collagen was visualized using a Masson’s Trichrome staining and analysis of interstitial fibrosis in the border zone region was performed semi-quantitative (scored 1–5; 1 = no interstitial fibrosis, 5 = excessive interstitial fibrosis) by two researchers blinded for group assessment.

### Mouse Efficacy Study

#### Anesthesia, Analgesia, and Surgical Procedure

Anesthesia was induced by intraperitoneal injection of medetomidine hydrochloride (1.0 g/kg body weight), midazolam (10.0 mg/kg), and fentanyl (0.1 mg/kg). Mice were intubated and connected to a respirator with a 1:1 oxygen-air ratio. During surgery, a core body temperature of 37°C was maintained by continuous rectal temperature monitoring and an automatic heating blanket. After a left lateral thoracotomy with an incision of the pericardium, the left coronary artery was ligated permanently with an 8–0 Ethilon suture (Ethicon). Ischemia was confirmed by bleaching of the myocardium and tachycardia, and surgical wounds were closed. Atipamezole hydrochloride (3.3 mg/kg), flumazenil (0.5 mg/kg) and buprenorphine (0.15 mg/kg) was used as an antagonist, and injected s.c. The evening of the day of operation and 12 h thereafter, s.c. injection of buprenorphine (0.15 mg/kg) was administered as analgesia.

#### Treatment

Prior to permanent ligation, mice were randomly assigned to receive either placebo (100 μL saline) or continued exenatide/potassium canrenoate treatment *i.v.* (continuous high dose, 0.15 μg/kg exenatide and 1 mg/kg of potassium canrenoate). *I.v.* treatment was continued 2 times daily for two consecutive days. On day 3, animals were anesthetized by isoflurane and small incisions were made in the skin. Osmotic mini-pumps containing either saline or exenatide and potassium canrenoate were implanted on the flank of the animal, providing active release of the compounds until termination. In order to prevent mixing of the therapeutics, two mini-pumps were randomly implanted on either side of the animal.

#### Echocardiography

At baseline, 7, 14, and 28 days after permanent ligation, all mice underwent echocardiography to assess cardiac geometry and function. Anesthesia was induced by inhalation of 2.0% isoflurane in a mixture of oxygen/air (1:1). Heart rate, respiration, and rectal temperature were constantly monitored and body temperature was kept between 36.0 and 38.0°C using heat lamps. Respiration gating, a 3D motor, and trigger points were used to obtain 300 transversal images of the heart during the expiratory phase, either at end-systole or end-diastole for complete 3D reconstruction of the heart. Image acquisition and analyses were performed using the Vevo 2,100 System and Software (Fujifilm VisualSonics Inc., Toronto, Canada).

#### Histological Analysis

Paraffin embedded hearts were cut in 3 µm thick sections. Before staining, the sections are deparaffinized [2 × 10 min in Ultraclear (1,466, Sakura), 2 × 5 min in 100% EtOH, (4099.9005 Klinipath), 2 × 5 min in 96% EtOH (Klinipath), 2 × 5 min in 70% EtOH (Klinipath), 5 min in Demi H_2_O]. Collagen was visualized using a Masson’s Trichrome staining and analysis of interstitial fibrosis in the border zone region was performed semi-quantitative (scored 1–5; 1 = no interstitial fibrosis, 5 = excessive interstitial fibrosis) by two researchers blinded for group assessment.

#### Statistics

All analyses were performed in a blinded, randomized fashion. Data are presented as mean ± SD. A one-way ANOVA was used to test any differences between the four porcine treatment groups. For the mice data, a mixed-models was used for repeated measurements [EDV, ESV, ejection fraction (EF)], with a random intercept for each mouse and as fixed factors group and time point. To determine whether the time course of the parameters was different for the groups, the interaction group*time point of measurement was also taken into the model. Heart weight/tibia length was analyzed with a student’s t-test. Statistical analyses were performed using SPSS and GraphPad Prism 8.3. A *p* ≤ 0.05 was considered statistically significant.

## Results

### Survival of Porcine Animals After Ischemia-Reperfusion Injury and Treatment With Exenatide/Potassium Canrenoate Combination Therapy

**FIGURE 1:**
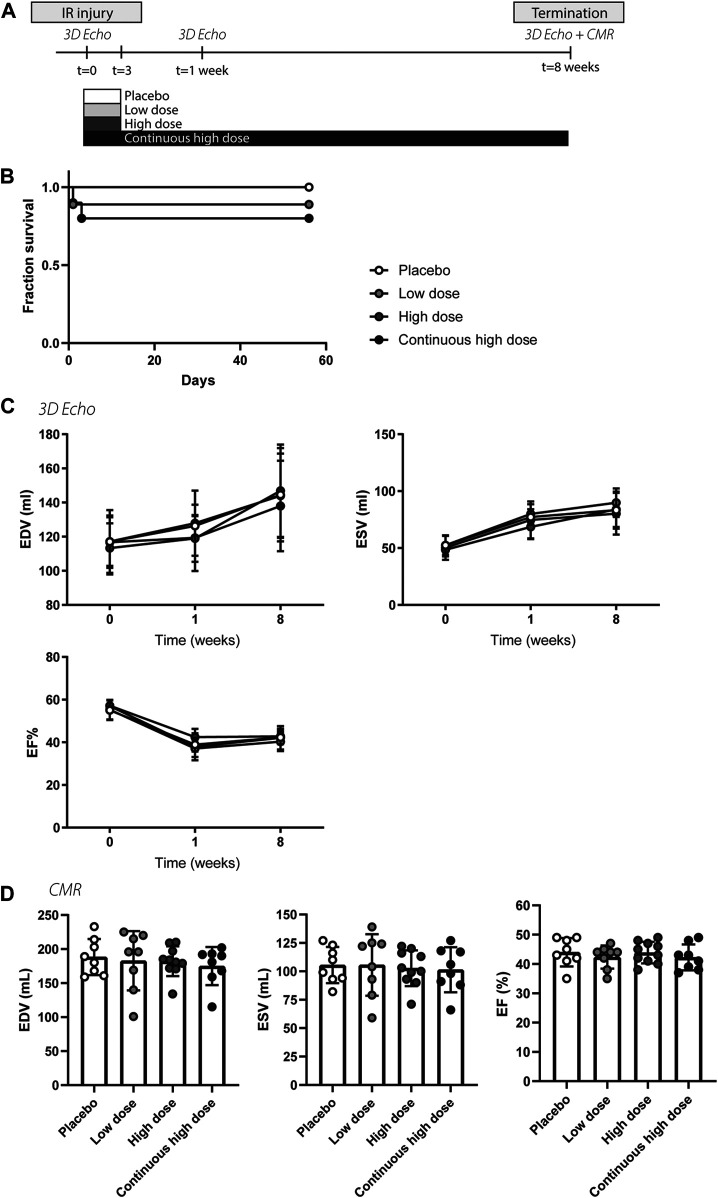
Study design, survival, and global cardiac function of porcine animals after severe ischemia-reperfusion injury. **(A)** Overview of the porcine study. **(B)** Fraction of survival of the different treatment groups, placebo, low dose, high dose, continuous high dose over the entire follow-up. IR, ischemia-reperfusion. **(C)** Similar end-systolic volume (ESV), end-diastolic volume (EDV), and ejection fraction (EF%) at baseline, 1 and 8 weeks post-IR measured by 3D echocardiography between different experimental groups. **(D)** ESV, EDV, and EF% at 8 weeks post-IR measured by cardiac magnetic resonance imaging did not differ between groups.

An overview of the study can be found in Figure 1A. A total of 40 porcine animals (69.2 ± 4.2 kg) were subjected to IR for 150 min. Nearly all animals developed ventricular arrhythmia during the occlusion period. Three animals died due to refractory VF not amenable by defibrillation before group assignment and another three animals during the follow-up period (two animals died on day IR + 1, one died on day IR + 3), presumably due to late VF (Figure 1B). This resulted in a total of 34 pigs completing the study and allowed a comparison of eight pigs in the placebo group, eight pigs in the low dose (0.05 μg/kg exenatide and 1 mg/kg of potassium canrenoate) group, 10 pigs in the high dose (0.15 μg/kg exenatide and 1 mg/kg of potassium canrenoate) group and eight pigs in the continuous high dose (0.15 μg/kg exenatide and 1 mg/kg of potassium canrenoate) group (Supplementary Table 1). Heart rate and mean arterial pressure was similar during the first hours after IR and compound administration (Supplementary Table 2).

### Exenatide/Potassium Canrenoate Combination Therapy Does not Influence Cardiac Function and Infarct Size in a Severe Porcine IR-Model

To validate the cardio-protective effect of exenatide/potassium canrenoate combination therapy on myocardial injury and dysfunction following severe IR, left ventricular performance was assessed by 3D-TEE and CMR. Baseline cardiac function (EDV, ESV, and EF) determined by echocardiography was similar in all groups (Figure 1C + Supplementary Figure 1). After 1 week, ESV significantly increased and EF significantly decreased in all four groups (*p* < 0.05, Figure 1C), indicative of successful infarct induction. Both at 1 and 8 weeks post-MI, no significant differences were observed between the four groups in end-diastolic and end-systolic volume nor in EF (Figure 1C + Supplementary Figure 1). Assessment of cardiac volumes and function measured with CMR after 8 weeks also showed no significant differences (Figure 1D). In summary, measurements of myocardial volumes and function were similar in all three treatment groups compared to the controls.

### Scar Size and Interstitial Cardiac Fibrosis is not Affected by Exenatide/Potassium Canrenoate Combination Therapy

**FIGURE 2:**
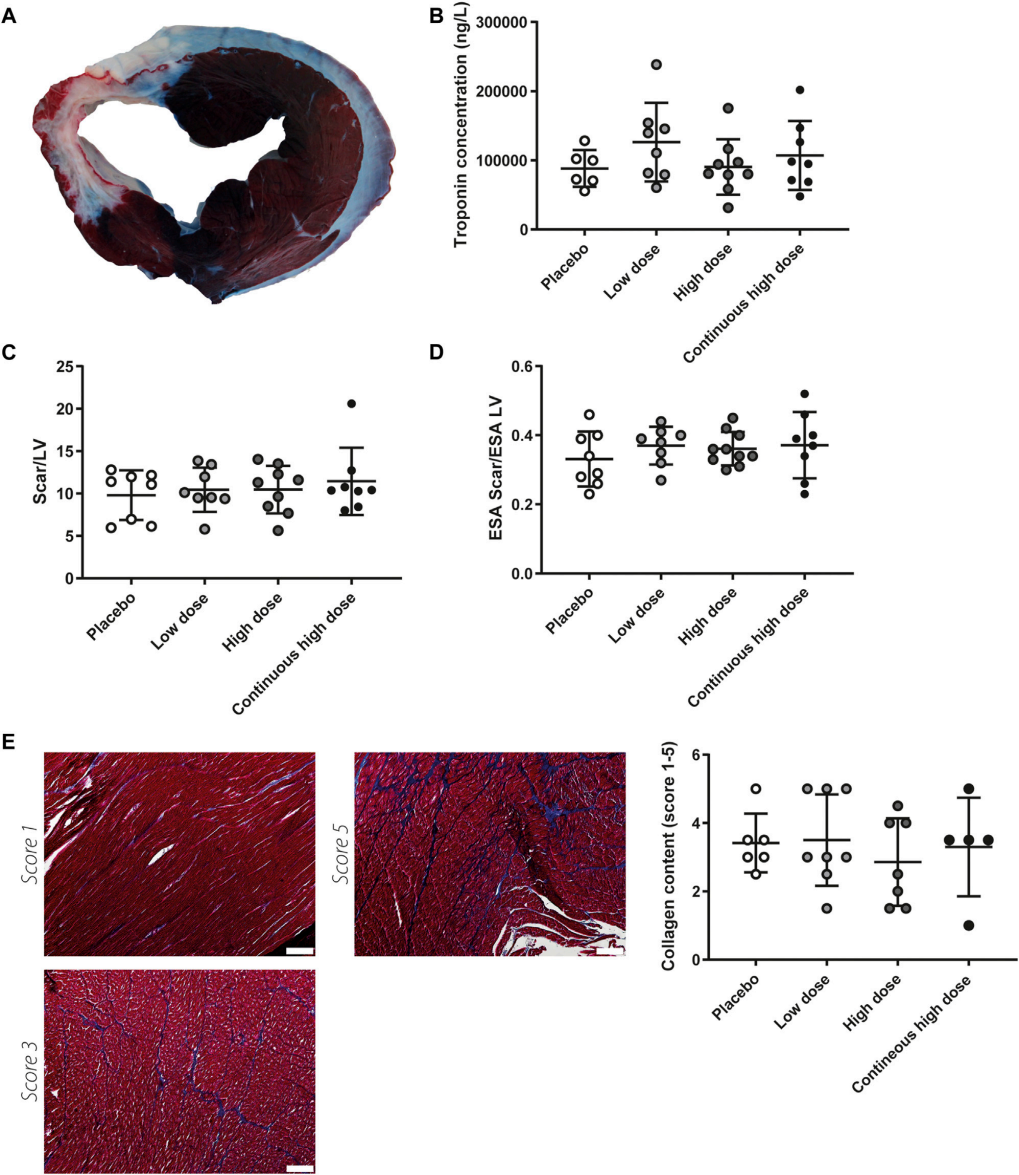
Myocardial damage and after severe ischemia-reperfusion injury. **(A)** Representative picture of myocardial slice; the dark area represents the remote area, white area represents infarcted myocardium. **(B)** Circulating levels of cardiac troponin 24 h after severe ischemia-reperfusion injury. No significant differences were observed between the different experimental groups. **(C)** Infarcted areas did not differ between treatment groups when expressed relative to measurements left ventricle (LV) at 8 weeks follow-up. **(D)** Infarct area as a fraction of LV endocardial surface area (ESA) was not different between treatment groups at 8 weeks follow-up. **(E)** Representative pictures of collagen content score 1, score 3, and score 5 after staining with Masson’s Trichrome, quantification showed no differences in interstitial fibrosis in the border zone region. Scale bar = 200 µm.

Solo treatment of exenatide has shown to reduce infarct size in a previous porcine MI study performed by our group (Timmers et al., 2009). As a direct reflection of cardiac damage, systemic troponin I levels were measured 24 h after IR. Analysis revealed troponin I levels did not differ between groups, suggesting infarct size is not affected by the combination therapy (Figure 2B). Accordingly, infarcted area/LV ratios, assessed by TTC staining, were not significantly different between the groups at 8 weeks follow-up (Figures 2A,C). Scar size was also analyzed as a fraction of the endocardial surface area (ESA) of the LV. Yet, scar size as a percentage of ESA was not significantly different among treatment groups (Figure 2D). As atrophy of the infarcted area, and hypertrophy of the remote endocardium may have influenced infarct size determination by TTC staining we additionally performed trichrome staining to visualize scar tissue. Histological analysis for interstitial fibrosis also showed no differences between groups (Figure 2E).

### Survival of Mice After Permanent Coronary Artery Ligation and Treatment With Exenatide/Potassium Canrenoate Combination Therapy

**FIGURE 3:**
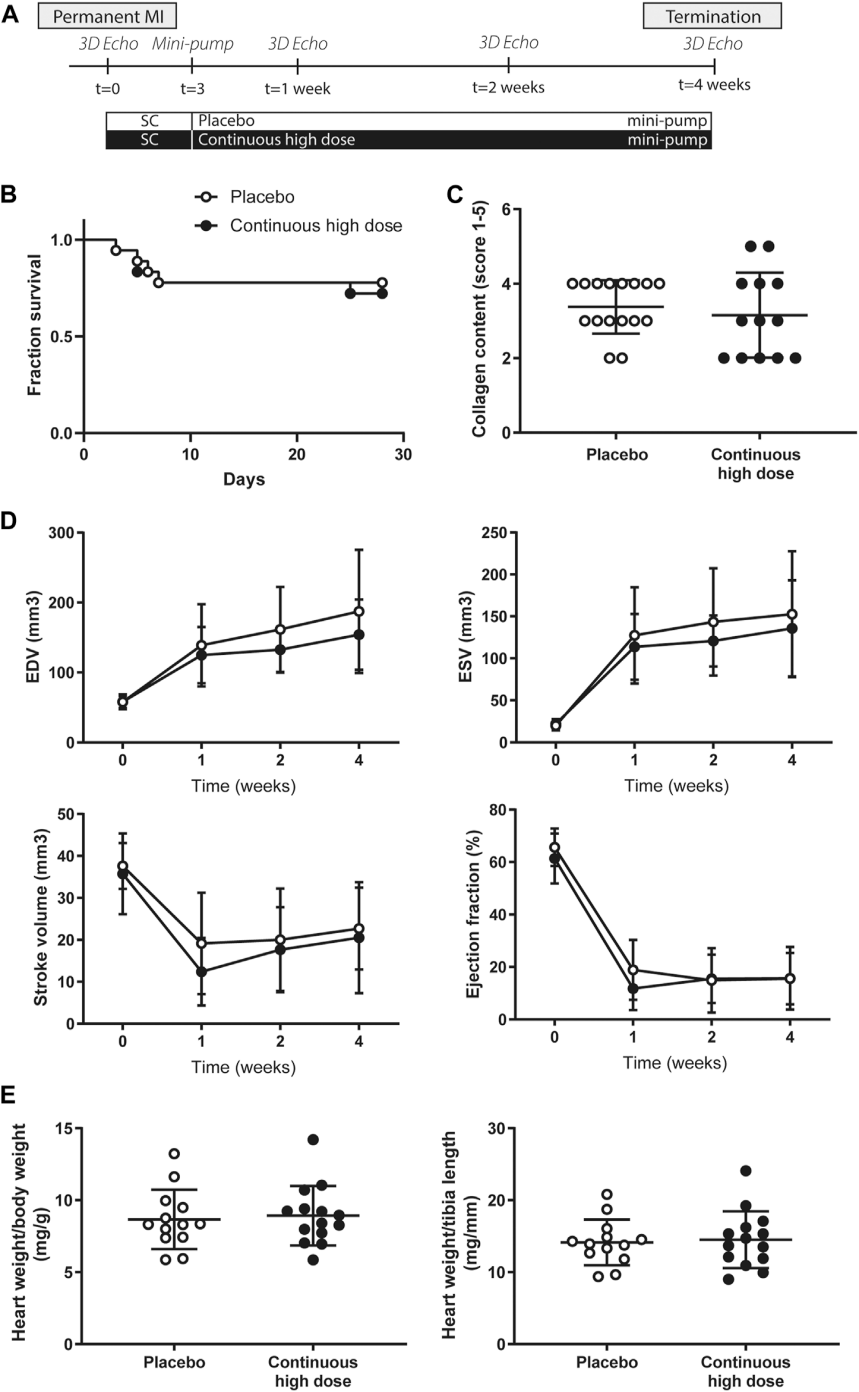
Study design, survival, global cardiac function and cardiac hypertrophy assessment in mice after permanent myocardial infarction. **(A)** Overview of the mice study. **(B)** Fraction of survival of placebo and continuous high dose treated animals over the entire follow-up. MI, myocardial infarction. **(C)** Quantification of collagen content score (1: no collagen—5: extensive collagen) showed no differences in interstitial fibrosis between placebo and continuous high dose treated animals. **(D)** End-systolic volume (ESV), end-diastolic volume (EDV), stroke volume (SV), and ejection fraction (EF) measured by 3D echocardiography at baseline, 7, 14, and 28 days after permanent ligation. No significant differences were observed between the different experimental groups. **(E)** Heart weight/body weight ratio (mg/g) and heart weight/tibia length (mg/mm) between groups were similar 28 days after permanent ligation.

To fully exclude potential effects of the compounds on IR injury and enabling to solely focus on adverse remodeling we tested the effects of continuous administration of exenatide and potassium canrenoate on myocardial function and cardiac remodeling in a severe mouse model of MI with permanent ligation. An overview of the mice study can be found in Figure 3A. A total of 36 animals were subjected to permanent LAD ligation. Within 28 days after permanent ligation, nine animals died in total, of which 2 as a direct consequence of the surgery (2 days post-surgery). Other animals died during follow-up (tree animals on day 5, one animal on day 6, two animals on day 7 and one animal on day 25 post ligation) (Figure 3B), but without differences between the groups. This resulted in a total of 27 mice completing the study and allowed a comparison of 13 mice in the placebo group and 14 mice in the continuous high dose (0.15 μg/kg exenatide and 1 mg/kg of potassium canrenoate) group (Supplementary Table 3).

### Neither Cardiac Function nor Cardiac Hypertrophy is Influenced by Exenatide/Potassium Canrenoate Combination Therapy in Mice After Permanent Coronary Artery Ligation

At baseline, 7, 14, and 28 days post-MI, cardiac function was assessed by use of high-frequency 3D ultrasound. Baseline cardiac function (EDV, ESV, SV, and EF) was similar in all groups (Figure 3D). As a consequence of the permanent ligation, EDV and ESV increased significantly over time in both groups (Figure 3D, *p* < 0.05), while no significant differences were observed between the groups (Figure 3D + Supplementary Figure 2). Correspondingly, stroke volume and ejection fraction showed a significant decrease over time in both groups after induction of the myocardial infarction (Figure 3D, *p* < 0.05), but was not different between placebo and treated animals (Figure 3D, Supplementary Figure 2). At the end of the follow-up period, heart weight to body weight ratio and heart weight to tibia length (Figure 3E) was comparable in both groups indicating no differences in cardiac hypertrophy between the groups. Histological analysis for interstitial fibrosis also showed no differences between groups (Figure 3B).

## Discussion

In the current study, we hypothesized that combined treatment with GLP-1R agonist exenatide and MR antagonist potassium canrenoate could minimize cardiac injury and limit progression to chronic HF. By using different treatment arms in the pig model we could discriminate between the effect of the combination therapy administered in the early phase after severe IR and long-term administration on adverse cardiac remodeling. Treatment in the acute phase with two different doses of combination therapy did not show effects on cardiac function, scar size or interstitial fibrosis 8 weeks post severe IR injury. The same holds for chronic treatment with high-dose combination therapy. In addition, long-term administration with high dose combination therapy in a mouse model of permanent MI did not show any beneficial effects as well.

Post-MI-cardiac remodeling comprises pathophysiological interactions between cellular and extracellular components resulting in biochemical and metabolic changes, including formation of reactive oxygen species, inflammation, and abrupt changes in pH− and Ca2+ levels, contributing to apoptosis of cardiomyocytes and excessive fibrosis formation (Yellon and Hausenloy, 2007; Nielsen et al., 2019). Separate use of both compounds in different scenarios has shown to have beneficial effects targeting these changes. In an earlier study, we showed increased activity of antioxidant enzymes, reduced nuclear oxidative stress and fewer apoptotic cells in exenatide-treated porcine animals 3 days after IR injury (Timmers et al., 2009) and similar results were found with exenatide pre-treatment for 2 weeks prior to IR in a rat study (Chang et al., 2013). In addition, it was shown that exenatide pre-treatment improved morphological and mechanical changes of mitochondria in response to IR injury in a rat model (Lee et al., 2017). In all studies, these findings were accompanied with reduced infarct size and improved cardiac function compared to control-treated animals. MRAs have shown to reduce collagen accumulation and fibrosis in animal models with permanent MI (Wang et al., 2004; Takeda et al., 2007). In patients, potassium canrenoate treatment also significantly reduced post-infarction collagen synthesis measured by serum N-terminal propeptide of type III procollagen (PIIINP) levels which was accompanied by smaller ventricular volumes (Modena et al., 2001).

Although other combinations of compounds were used, combined treatment of exenatide and with dapagliflozin, a sodium-glucose co-transporter-2 (SGLT2) inhibitor, showed to improve markers associated with liver steatosis and fibrosis in type 2 diabetes patients compared to either exenatide or dapagliflozin administration alone (Gastaldelli et al., 2020). In addition, in rats with post-MI heart failure, a combination of canrenone, the active metabolite of potassium canrenoate and ramipril, angiotensin-converting enzyme (ACE) inhibitor, attenuated LV dilation and interstitial remodeling and improved cardiac function compared solo-treatment (Cittadini et al., 2003). Building on these results, exenatide, and potassium canrenoate combination therapy could have a synergistic effect on limiting IR injury and cardiac remodeling and thus progression to HF.

One may argue that administered concentrations in this study were not sufficient to realize any beneficial effects. However, the chosen dosing concentrations in this study were comparable with concentrations found in the literature. Apart from the initial administration prior to IR, pigs received the same dose of exenatide compared to a porcine study previously performed by our group (Timmers et al., 2009). During initial dosing, only IV administration was performed as in our study both exenatide and potassium canrenoate was given prior to IR. Potassium canrenoate dose was slightly higher compared to dosages used in clinical studies (Li et al., 2013) but matched the concentration that worked best in reducing infarct size and lowering circulating troponin levels in an acute study performed in mice and rabbits (Schmidt et al., 2010). Differences in pharmacokinetics between species due to differences in volume distribution, clearance, and absorption could be of influence (Frey et al., 1988). Whether this is the case for these compounds is not clear. Higher concentrations of potassium canrenoate (bolus of 200 mg in animals of ∼30 kg) were used in a pig model investigating the effect of potassium canrenoate during increased intraabdominal pressure (Gudmundsson et al., 2004). However, here only acute changes in renal hemodynamics and urinary output were investigated and no long-term assessment was carried out. To not risk overdosing or adverse effects in our studies, lower dosing was implemented. In addition, with the current study design, we cannot exclude that drug interaction of exenatide with potassium canrenoate resulted in a neutral result. To the best of our knowledge, no reports on adverse interactions between these drugs have been published, and given the completely different molecular pathways they target, we argue it is unlikely that interaction of the drugs could have contributed to the neutral results.

Apart from dose, the chosen models could play a role in the absence of effect. The rationale of this study was to assess the effects of combined exenatide and potassium canrenoate therapy on adverse cardiac remodeling solely and without affecting infarct size. Therefore, pigs were exposed to prolonged ischemia times and the mice were subjected to permanent ligation. By comparing 60, 75 and 90 min occlusion in the closed chest LAD ligation model in pigs it was recently shown that increasing occlusion times lead to significantly larger infarct sizes, with almost complete transmural infarcts upon 90 min occlusion time (Silvis et al., 2021) In the current study, we used a porcine model of severe IR injury with 150 min occlusion time and a mice model of permanent myocardial infarction. Consequential to the prolonged ischemia time the damage is presumably in an irreversible state, thereby limiting the therapeutic benefit on salvaging myocardial ischemia-reperfusion-related injury. Accordingly, the findings of these studies do not preclude the effects of the combination therapy on myocardial reperfusion injury in pigs with a shorter occlusion times. Although there were difficulties to translate these favorable outcomes to clinical beneficial effects (Roos et al., 2016), we indeed found exenatide can inhibit reperfusion injury in the earlier study performed with exenatide mono-treatment (Timmers et al., 2009). This may suggest exenatide primarily inhibits reperfusion driven injury which is in our severe models very limited or not even present at all. Further direct comparison between these studies is complicated due to methodological differences, including different location of coronary artery occlusion (LCx vs LAD), difference in ischemia time (75 vs. 150 min), time of follow-up (3 days vs. 8 weeks), and different methods to determine cardiac function (echo vs. CMR).

Although our mouse model with permanent ligation resulted in an extreme loss of function (mean EF of 16% at termination), a less but still severe decrease in cardiac function was observed in our porcine model of severe IR injury with 150 min occlusion time (mean EF of 41% at termination). Whether the combination therapy has an effect on pigs with even more severe heart failure (EF < 35%) remains unclear and is difficult to investigate due to low survival rates when occluding the LAD more proximal.

Cardiac remodeling is quickly initiated in response to ischemia. While this remodeling process is focused on wound healing and proper scar formation in the first few days, post-MI progressive collagen deposition in the non-infarcted area is initiated after a few days thereby contributing to late adverse cardiac remodeling and HF progression (Nielsen et al., 2019). Many of the beneficial effects of GLP-1R agonist and MRAs in preclinical models are shown in the acute setting (i.e. short follow-up time) (Timmers et al., 2009; Schmidt et al., 2010; Chang et al., 2013; Roos et al., 2016). In clinical data, treatment with canrenoate did not show any significant differences at entry and at 10 days in echocardiographic parameters such as EDV, ESV, and EF, while at 90 days, and 180 days after treatment it did (Di Pasquale et al., 2005). The same was observed in another patient study with potassium canrenoate (Modena et al., 2001). Here, also collagen synthesis marker PIIINP illustrated a more profound decrease over time (3, 6, 12 months). Evidently, beneficial functional effects can be observed when targeting late cardiac remodeling. Therefore, it may be possible that the timing of our study (8 weeks follow-up) was just outside the optimal therapeutic window: too late to benefit acute-, and too early to benefit long-term effects.

In the current study, combined treatment with GLP-1R agonist exenatide and MR antagonist potassium canrenoate did not show functional benefits on cardiac remodeling and did not result in functional improvement in small and large chronic HF models. Yet, the role of either compound in IR injury and HF progression remains appealing and more studies are necessary to provide complete disclosure concerning their role in this process.

## Data Availability

The raw data supporting the conclusion of this article will be made available by the authors, without undue reservation.

## References

[B1] Bhatt A. S. Ambrosy A. P. Velazquez E. J. (2017). Adverse Remodeling and Reverse Remodeling after Myocardial Infarction. Curr. Cardiol. Rep. 19 (8), 71. 10.1007/s11886-017-0876-4 28660552

[B2] Brilla C. G. Weber K. T. (1992). Mineralocorticoid Excess, Dietary Sodium, and Myocardial Fibrosis. J. Lab. Clin. Med. 120 (6), 893–901. 1453111

[B3] Chang G. Zhang D. Yu H. Zhang P. Wang Y. Zheng A. (2013). Cardioprotective Effects of Exenatide against Oxidative Stress-Induced Injury. Int. J. Mol. Med. 32 (5), 1011–1020. 10.3892/ijmm.2013.1475 23982489

[B4] Cittadini A. Monti M. G. Isgaard J. Casaburi C. Stromer H. Di Gianni A. (2003). Aldosterone Receptor Blockade Improves Left Ventricular Remodeling and Increases Ventricular Fibrillation Threshold in Experimental Heart Failure. Cardiovasc. Res. 58 (3), 555–564. 10.1016/s0008-6363(03)00251-7 12798428

[B5] Di Pasquale P. Cannizzaro S. Scalzo S. Parrinello G. Fasullo S. Giambanco F. (2005). Effects of Canrenoate Plus Angiotensin-Converting Enzyme Inhibitors versus Angiotensin-Converting Enzyme Inhibitors Alone on Systolic and Diastolic Function in Patients with Acute Anterior Myocardial Infarction. Am. Heart J. 150 (5), e1–919. 10.1016/j.ahj.2005.03.032 16290961

[B6] Ellenbroek G. H. J. M. van Hout G. P. J. Timmers L. Doevendans P. A. Pasterkamp G. Hoefer I. E. (2016). Primary Outcome Assessment in a Pig Model of Acute Myocardial Infarction. J. Vis. Exp. 116, 54021. 10.3791/54021 PMC5092202 27768034

[B7] Frey B. M. Sieber M. Mettler D. Gänger H. Frey F. J. (1988). Marked Interspecies Differences between Humans and Pigs in Cyclosporine and Prednisolone Disposition. Drug Metab. Dispos 16 (2), 285–289. 2898348

[B8] Gastaldelli A. Repetto E. Guja C. Hardy E. Han J. Jabbour S. A. (2020). Exenatide and Dapagliflozin Combination Improves Markers of Liver Steatosis and Fibrosis in Patients with Type 2 Diabetes. Diabetes Obes. Metab. 22 (3), 393–403. 10.1111/dom.13907 31692226 PMC7064910

[B9] Gudmundsson F. F. Viste A. Myking O. L. Grong K. Svanes K. (2004). Effects of the Aldosterone Receptor Antagonist Potassium Canrenoate on Renal Blood Flow and Urinary Output during Prolonged Increased Intraabdominal Pressure (IAP) in Pigs. Surg. Endosc. 18 (10), 1528–1534. 10.1007/s00464-003-9295-2 15791383

[B10] Lee K. H. Ha S. J. Woo J.-S. Lee G.-J. Lee S.-R. Kim J. W. (2017). Exenatide Prevents Morphological and Structural Changes of Mitochondria Following Ischaemia-Reperfusion Injury. Heart Lung Circ. 26 (5), 519–523. 10.1016/j.hlc.2016.08.007 27743854

[B11] Li X. Qi Y. Li Y. Zhang S. Guo S. Chu S. (2013). Impact of Mineralocorticoid Receptor Antagonists on Changes in Cardiac Structure and Function of Left Ventricular Dysfunction. Circ. Heart Fail. 6 (2), 156–165. 10.1161/CIRCHEARTFAILURE.112.000074 23400891

[B12] Lønborg J. Vejlstrup N. Kelbæk H. Bøtker H. E. Kim W. Y. Mathiasen A. B. (2012). Exenatide Reduces Reperfusion Injury in Patients with ST-Segment Elevation Myocardial Infarction. Eur. Heart J. 33 (12), 1491–1499. 10.1093/eurheartj/ehr309 21920963

[B13] Modena M. G. Aveta P. Menozzi A. Rossi R. (2001). Aldosterone Inhibition Limits Collagen Synthesis and Progressive Left Ventricular Enlargement after Anterior Myocardial Infarction. Am. Heart J. 141 (1), 41–46. 10.1067/mhj.2001.111258 11136485

[B14] Nielsen S. H. Mouton A. J. DeLeon-Pennell K. Y. Genovese F. Karsdal M. Lindsey M. L. (2019). Understanding Cardiac Extracellular Matrix Remodeling to Develop Biomarkers of Myocardial Infarction Outcomes. Matrix Biol. 75-76, 43–57. 10.1016/j.matbio.2017.12.001 29247693 PMC6002886

[B15] Noyan-Ashraf M. H. Momen M. A. Ban K. Sadi A.-M. Zhou Y.-Q. Riazi A. M. (2009). GLP-1R Agonist Liraglutide Activates Cytoprotective Pathways and Improves Outcomes after Experimental Myocardial Infarction in Mice. Diabetes 58 (4), 975–983. 10.2337/db08-1193 19151200 PMC2661586

[B16] Pitt B. Remme W. Zannad F. Neaton J. Martinez F. Roniker B. (2003). Eplerenone, a Selective Aldosterone Blocker, in Patients with Left Ventricular Dysfunction after Myocardial Infarction. N. Engl. J. Med. 348 (14), 1309–1321. 10.1056/NEJMoa030207 12668699

[B17] Pitt B. Zannad F. Remme W. J. Cody R. Castaigne A. Perez A. (1999). The Effect of Spironolactone on Morbidity and Mortality in Patients with Severe Heart Failure. N. Engl. J. Med. 341 (10), 709–717. 10.1056/NEJM199909023411001 10471456

[B18] Read P. A. Khan F. Z. Dutka D. P. (2012). Cardioprotection against Ischaemia Induced by Dobutamine Stress Using Glucagon-like Peptide-1 in Patients with Coronary Artery Disease. Heart 98 (5), 408–413. 10.1136/hrt.2010.219345 21561896

[B19] Roos S. T. Timmers L. Biesbroek P. S. Nijveldt R. Kamp O. van Rossum A. C. (2016). No Benefit of Additional Treatment with Exenatide in Patients with an Acute Myocardial Infarction. Int. J. Cardiol. 220, 809–814. 10.1016/j.ijcard.2016.06.283 27394978

[B20] Schmidt K. Tissier R. Ghaleh B. Drogies T. Felix S. B. Krieg T. (2010). Cardioprotective Effects of Mineralocorticoid Receptor Antagonists at Reperfusion. Eur. Heart J. 31 (13), 1655–1662. 10.1093/eurheartj/ehp555 20028693 PMC3063847

[B21] Schmidt M. Jacobsen J. B. Lash T. L. Botker H. E. Sorensen H. T. (2012). 25 Year Trends in First Time Hospitalisation for Acute Myocardial Infarction, Subsequent Short and Long Term Mortality, and the Prognostic Impact of Sex and Comorbidity: a Danish Nationwide Cohort Study. BMJ 344, e356. 10.1136/bmj.e356 22279115 PMC3266429

[B22] Silvis M. J. M. van Hout G. P. J. Fiolet A. T. L. Dekker M. Bosch L. van Nieuwburg M. M. J. (2021). Experimental Parameters and Infarct Size in Closed Chest Pig LAD Ischemia Reperfusion Models; Lessons Learned. BMC Cardiovasc. Disord. 21 (1), 171. 10.1186/s12872-021-01995-7 33845779 PMC8042863

[B23] Takeda M. Tatsumi T. Matsunaga S. Hayashi H. Kimata M. Honsho S. (2007). Spironolactone Modulates Expressions of Cardiac Mineralocorticoid Receptor and 11.BETA.-Hydroxysteroid Dehydrogenase 2 and Prevents Ventricular Remodeling in Post-Infarct Rat Hearts. Hypertens. Res. 30 (5), 427–437. 10.1291/hypres.30.427 17587755

[B24] Timmers L. Henriques J. P. S. de Kleijn D. P. V. Devries J. H. Kemperman H. Steendijk P. (2009). Exenatide Reduces Infarct Size and Improves Cardiac Function in a Porcine Model of Ischemia and Reperfusion Injury. J. Am. Coll. Cardiol. 53 (6), 501–510. 10.1016/j.jacc.2008.10.033 19195607

[B25] Wang D. Liu Y.-H. Yang X.-P. Rhaleb N.-E. Xu J. Peterson E. (2004). Role of a Selective Aldosterone Blocker in Mice with Chronic Heart Failure. J. Card. Fail. 10 (1), 67–73. 10.1016/s1071-9164(03)00578-5 14966777

[B26] Yellon D. M. Hausenloy D. J. (2007). Myocardial Reperfusion Injury. N. Engl. J. Med. 357 (11), 1121–1135. 10.1056/NEJMra071667 17855673

[B27] Zannad F. McMurray J. J. V. Krum H. van Veldhuisen D. J. Swedberg K. Shi H. (2011). Eplerenone in Patients with Systolic Heart Failure and Mild Symptoms. N. Engl. J. Med. 364 (1), 11–21. 10.1056/NEJMoa1009492 21073363

