# Supplementary material for: Neutral Effects of Combined Treatment With GLP-1R Agonist Exenatide and MR Antagonist Potassium Canrenoate on Cardiac Function in Porcine and Murine Chronic Heart Failure Models
Source: Front Pharmacol. 2021 Jul 26;12:702326. doi: 10.3389/fphar.2021.702326 (PMC8352472; doi:10.3389/fphar.2021.702326)
Supplement: Supplementary file 1 [file DataSheet1.PDF]

Supplemental figure legends and tables

Supplemental figure 1. Cardiac function of individual porcine animals measured by 3D echocardiography.

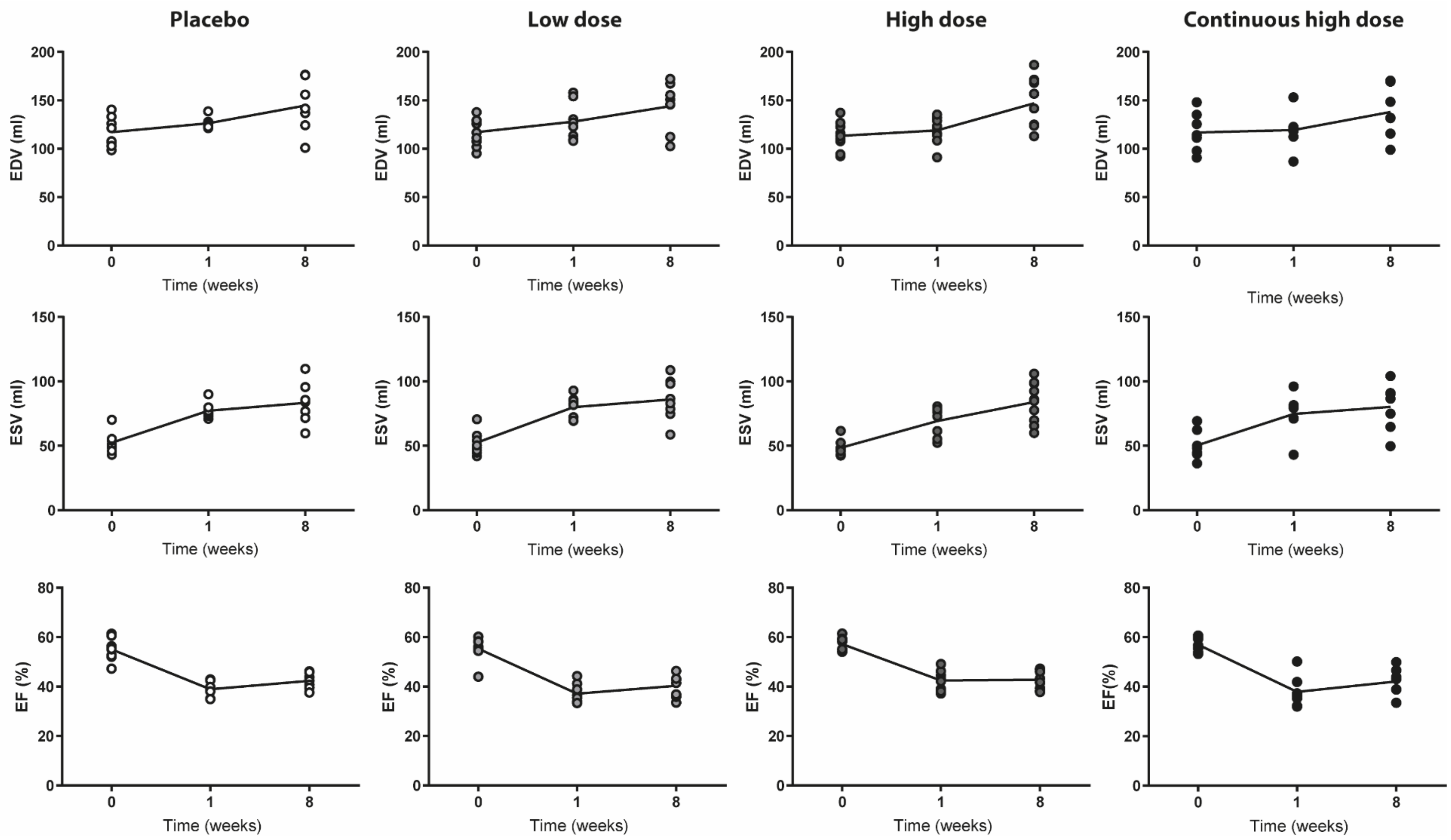

Individual values of end-systolic volume (ESV), end-diastolic volume (EDV) and ejection fraction (EF%) at baseline, 1 week and 8 weeks after severe ischemia-reperfusion injury.

Supplemental figure 2. Cardiac function of individual mice measured by 3D echocardiography.

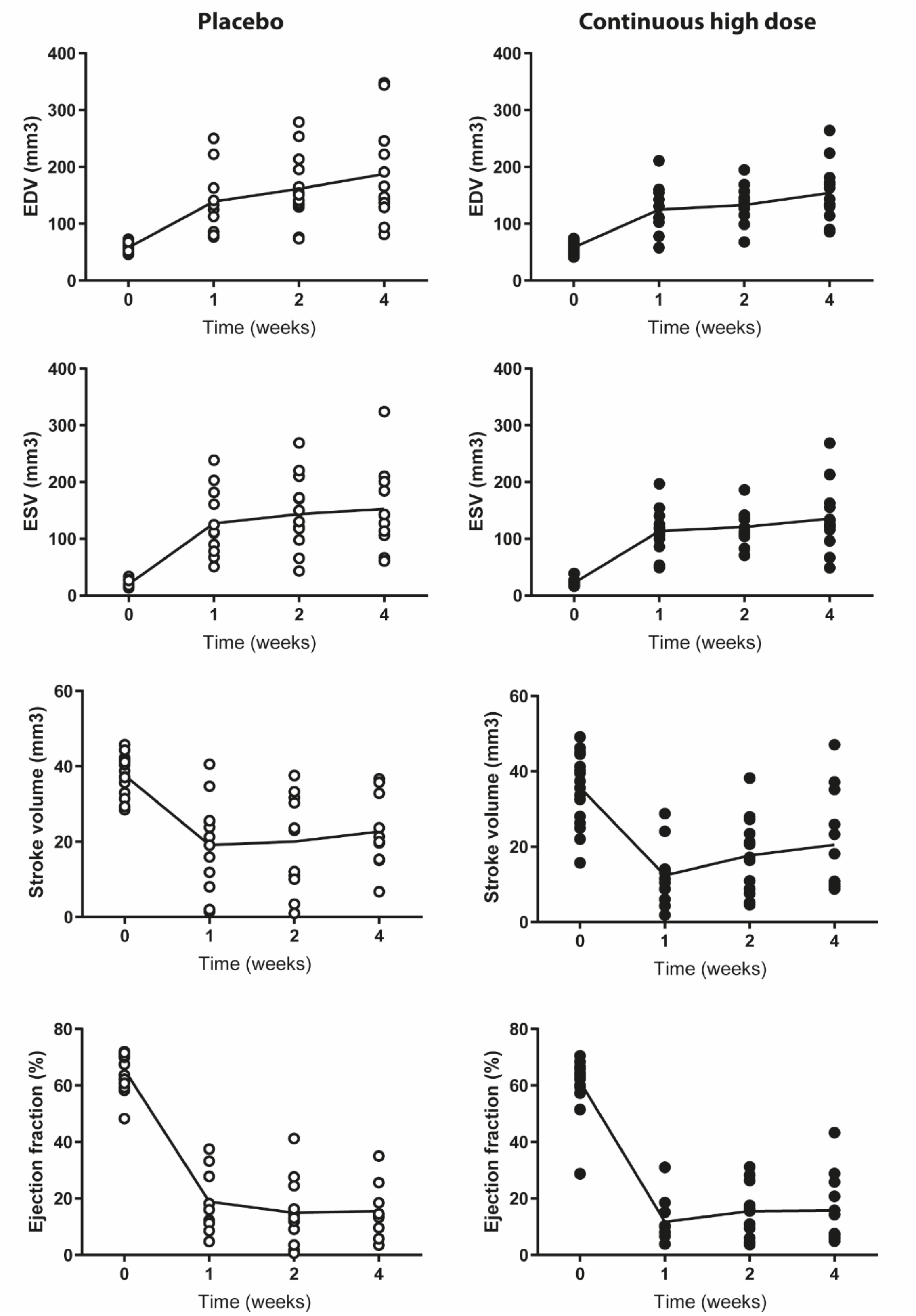

Individual values of end-systolic volume (ESV), end-diastolic volume (EDV), stroke volume (SV) and ejection fraction (EF%) at 7 days, 14 days and 28 days after permanent ligation.

Supplemental table 1. Overview of the treatment arms in the pig study

Group	Day 0	Day 1	Day 2	Day 3 - termination
**Placebo (n=8)**<br>saline	IV infusion prior IR	IV infusion 2x daily	IV infusion 2x daily	Treatment ended
**Treatment low dose (n=8)**<br>0.05ug/kg exenatide and 1mg/kg of potassium canrenoate	IV infusion prior IR	IV infusion 2x daily	IV infusion 2x daily	Treatment ended
**Treatment high dose (n=10)**<br>0.15ug/kg exenatide and 1mg/kg of potassium canrenoate	IV infusion prior IR	IV infusion 2x daily	IV infusion 2x daily	Treatment ended
**Treatment continuous high dose (n=8)**<br>0.15ug/kg exenatide and 1mg/kg of potassium canrenoate	IV infusion prior IR	IV infusion 2x daily	IV infusion 2x daily	IV infusion 2x daily

Abbreviations: IV, intravenous; IR, ischemia reperfusion

Supplemental table 2. Hemodynamic parameters during ischemia and reperfusion among different treatment arms (mean±SD).

	**Heart rate (bpm)**				**Mean arterial blood pressure (mmHg)**			
**Time**	***Placebo***	***Low dose***	***High dose***	***Continuous high dose***	***Placebo***	***Low dose***	***High dose***	***Continuous high dose***
**0**	61±4	57±8	63±12	53±8	106±19	91±17	94±16	88±12
**30**	65±8	65±13	64±22	61±8	103±37	81±22	84±19	85±19
**60**	63±10	62±12	59±10	74±42	106±21	87±22	84±27	89±37
**90**	65±12	64±16	61±10	65±12	100±11	78±31	92±30	100±28
**120**	64±7	66±12	64±14	58±11	95±11	97±18	94±26	106±23
**150**	76±31	65±10	81±12	88±26	91±14	85±9	98±17	103±22
**180**	94±15	93±14	102±12	98±20	80±12	80±14	83±14	86±26

Heart rate, displayed as bpm = beats per minute. Mean arterial blood pressure, displayed as mmHg = millimeters mercury

Supplemental table 3. Overview of the treatment arms in the mice study

Group	Day 0	Day 1	Day 2	Day 3 - termination
**Placebo (n=13)**<br>saline	IV prior ligation	IV 2x daily	IV 2x daily	Continuous infusion via osmotic minipump
**Treatment continuous high dose (n=14)**<br>0.15ug/kg exenatide and 1mg/kg of potassium canrenoate	IV prior ligation	IV 2x daily	IV 2x daily	Continuous infusion via osmotic minipump

Abbreviations: IV, intravenous
